# The Prognostic Impact of Cardiorespiratory Fitness on Mortality in Diabetes Patients With and Without Left Ventricular Hypertrophy

**DOI:** 10.31083/RCM48314

**Published:** 2026-06-29

**Authors:** Eric S Nylén, Shikha G Khosla, Charles Faselis, Andreas Pittaras, Monica Aiken, Peter Kokkinos

**Affiliations:** ^1^Division of Medicine, Section of Endocrinology, Veterans Affairs Medical Center, Washington, DC 20422, USA; ^2^Division of Medicine, Section of Endocrinology, George Washington University, Washington, DC 20052, USA; ^3^Division on Medicine, Veterans Affairs Medical Center, Washington, DC 20422, USA; ^4^George Washington University School of Medicine and Health Sciences, Washington, DC 20052, USA; ^5^Division of Medicine, Section of Cardiology, Veterans Affairs Medical Center, Washington, DC 20422, USA; ^6^Department of Kinesiology and Health, Rutgers University, New Brunswick, NJ 08901-8525, USA; ^7^Division of Medicine, Section of Cardiology, Georgetown University School of Medicine, Washington, DC 20007, USA

**Keywords:** type 2 diabetes, echocardiography, left ventricular hypertrophy, cardiorespiratory fitness, mortality

## Abstract

**Background:**

Left ventricular hypertrophy (LVH) and type 2 diabetes mellitus (T2DM) are both independent risk factors for mortality. Cardiorespiratory fitness (CRF) is inversely associated with mortality and independently predicts lower mortality risk in T2DM. However, the relationship between CRF, LVH, and mortality risk in T2DM has not been well characterized and was the focus of this study.

**Methods:**

A total of 866 individuals with T2DM (mean age 61.6 ± 9.9 years) underwent both a standardized exercise stress test and an echocardiographic evaluation. We then established two fitness categories based on peak CRF (i.e., metabolic equivalents (METs)). Individuals with a peak MET level below the median (<6 METs) were considered Low-Fit, and those with a peak MET level at or above the median (≥6 METs) were classified as fit. Left ventricular mass (LVM) was calculated using a standardized formula and indexed to body size to obtain the LVM index. To assess the interaction between fitness and LVH, we established four groups based on fitness status and the presence or absence of LVH: Low-Fit/No LVH (n = 225); Low-Fit/LVH (n = 236); Fit/No LVH (n = 218); and Fit/LVH (n = 187). The Low-Fit/No LVH group served as the reference category for all survival analyses.

**Results:**

Over a total of 24 years of follow-up (median 8.9 years), 346 deaths occurred, corresponding to an annual mortality rate of 4.3% in the entire cohort. In the Cox proportional hazards analysis, models adjusted for age, body mass index (BMI), hypertension, smoking, and medication use, revealed a 20% higher mortality risk in the Low-Fit/LVH group (hazard ratio (HR): 1.20; 95% confidence interval (CI): 0.93–1.56; *p* = 0.15). In contrast, mortality risk was 41% lower in the Fit/No LVH individuals (HR: 0.59; 95% CI: 0.42–0.82; *p* = 0.002) and 43% lower in the Fit/LVH individuals (HR: 0.57; 95% CI: 0.40–0.81; *p* = 0.002).

**Conclusions:**

Low-Fit individuals with LVH showed a trend toward higher mortality risk. This risk was significantly mitigated in individuals with moderate fitness regardless of LVH status.

## 1. Introduction

Type 2 diabetes mellitus (T2DM) is a ubiquitous, escalating, and costly public health problem. The latest Center for Disease Control (CDC) data reveal that 38.4 million subjects had diabetes in 2021 which represents 11.6% of the US population [1]. Moreover, the main drivers of T2DM, i.e., obesity, prediabetes, and aging, are widespread health issues: 97.6 million adults (≥18 years old) have prediabetes, and approximately three-quarters of those at or above 25 years old are either overweight or obese [2,3]. Importantly, patients with diabetes are at a significantly increased risk for cardiovascular disease (CVD) and its associated morbidity and mortality [4].

Left ventricular hypertrophy (LVH) identified by echocardiography independently predicts cardiovascular morbidity and mortality [5,6,7]. Left ventricular hypertrophy is also a major predictor of morbidity and mortality in the general population [8,9]. Notably, LVH significantly reclassifies risk when added to CV risk factors in most [10], although not all studies [11]. Left ventricular hypertrophy in T2DM patients is highly prevalent but typically difficult to detect clinically [12,13,14]. In one study, the presence of T2DM increased the risk of LVH by approximately 1.5-fold [15]. In the Strong Heart Study of American Indians, T2DM individuals showed significantly higher LV mass and wall thicknesses but reduced LV fractional shortening [16]. In another study of a relatively diverse and healthy population, T2DM was associated with increased LV mass and decreased LV mid-wall function, which may contribute to the high rates of overt coronary heart disease [17]. Moreover, the effects of diabetes on LV mass are magnified by interactions with obesity and aging [18].

Mechanistically, transient ischemic dilation of the LV has been associated with poor prognosis which is linked to diabetes, LVH, or both [19]. Additionally, insulin and its signaling pathways have pleiotropic effects on heart function; insulin resistance, hyperinsulinemia, and hyperglycemia—hallmarks of T2DM—may play maladaptive roles leading to LVH and impaired cardiorespiratory fitness (CRF) [20,21,22,23,24]. Indeed, the diabetic milieu is directly associated with LVH, as demonstrated in heart transplant recipients [25].

Poor CRF is a well-established independent predictor of cardiovascular and overall mortality among both healthy individuals and those with T2DM [26,27]. Moreover, the association between fitness and T2DM has been shown to be causal in Mendelian randomization studies [28]. Importantly, increased physical activity and higher CRF are associated with lower mortality in individuals with T2DM [29], with reductions in risk proportional to fitness levels. Improvements in CRF achieved by moderate-intensity physical activity may augment hemodynamics and cardiac performance in prehypertensive individuals, ultimately reducing LV mass [30,31]. Despite these findings, there is a paucity of data on the potential benefits of fitness among individuals with T2DM and LVH. Furthermore, the association between CRF assessed objectively by a standardized exercise tolerance test (ETT), LVH, and mortality risk in patients with T2DM has not been adequately explored and was therefore the aim of this study.

## 2. Research Design and Methods

### 2.1 Participants

A symptom-limited ETT and echocardiographic evaluation were administered at the Veterans Affairs Medical Center, Washington, DC, either as part of routine evaluations or to assess exercise-induced ischemia. This data along with the individual’s medical history was electronically stored. We identified those with non-insulin using T2DM using International Classification of Disease (ICD) coding. We excluded women and those with any of the following: (1) history of an implanted pacemaker, (2) left bundle branch block, (3) unable to complete the ETT, (4) ETT with evidence suggestive of ischemia, (5) impaired chronotropic response, and (6) those with HIV.

**Fig. 1.**
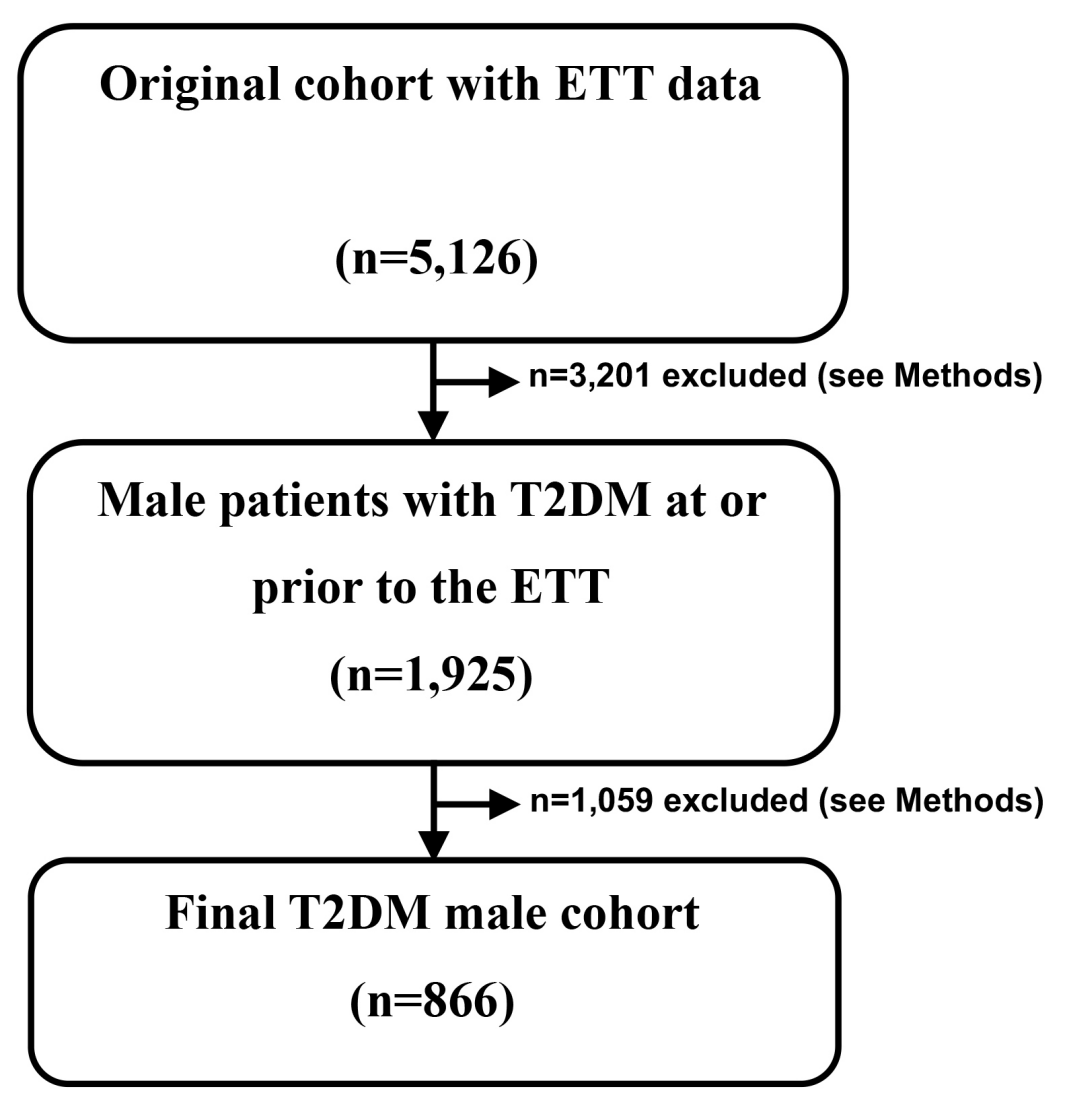
**Flowchart for the selection of patients**. ETT, exercise tolerance test; T2DM, type 2 diabetes mellitus.

After these exclusions, 866 men with T2DM (mean age 61.6 ± 9.9 years) were identified (Fig. 1). The institutional review board at our institution approved the study, and all subjects gave written informed consent before undergoing these studies. All demographic, clinical, and medication information was obtained from the subject’s computerized medical records just before their ETT and verified.

Dates of death were verified from the VA Beneficiary Identification and Record Locator System File. This system is used to determine benefits to survivors of veterans and has been shown to be 95% complete and accurate [32].

### 2.2 Exercise Assessments

Cardiorespiratory fitness was established by a standardized treadmill test using the Bruce protocol. Peak exercise capacity in metabolic equivalents (METs) was estimated by standardized equations based on peak treadmill speed and grade [33]. Subjects were encouraged to exercise until volitional fatigue in the absence of symptoms or other indications for stopping [34]. The use of handrails was discouraged but allowed when necessary for balance and safety. Age-predicted peak exercise heart rate was determined on the basis of a population-specific equation [35]. Medications were not altered before testing.

### 2.3 Echocardiographic Evaluations

All of the echocardiographic studies were analyzed by a qualified cardiologist who was blinded to the results of the ETT. Left ventricular systolic dimension and left ventricular diastolic dimensions (LVDDs), inter-ventricular septal (IVS) thickness, and posterior wall (PW) thickness were measured following the guidelines of the American Society of Echocardiography: Left ventricular mass was computed with the Devereux equation (left ventricular mass (LVM) = 0.8 × [1.04 × (IVS + PW + LVDD)^3^ – (LVDD)^3^] + 0.6. LVM was then indexed to body size by dividing raw LVM by height in meters to the allometric power of 2.7 to obtain LVM index. LVH was defined as LVM index >48 g/m^2.7^) [36,37] and indexed for body surface area (DuBois formula). Moreover, we excluded those patients with severe valvular disease (i.e., severe aortic stenosis, or those with moderate/severe mitral regurgitation), known hypertrophic cardiomyopathy, large transmural myocardial infarctions, aortic aneurysm, and neoplastic or systemic disease expected to limit patient’s life expectancy. Patients with diagnosis of cardiac amyloidosis (ICD-9 code 277.39) were also excluded.

The M-mode, 2-dimensional, and Doppler echocardiographic examinations were performed by trained technologists, and read by experienced senior echocardiographers [38].

### 2.4 Determination of the Categories Based on the LVH Status and the Fitness

We determined two fitness categories based on peak exercise capacity (METs) achieved. Individuals with a peak MET level below the median (<6 METs) were considered Low-Fit and those at or above the median (≥6 METs) were considered Fit [29].

To assess the risk associated by the interaction between fitness status and LVH we established a total of four groups: (1) Low-Fit/No LVH (n = 225); (2) Low-Fit/LVH (n = 236); (3) Fit/No LVH (n = 218); and (4) Fit/LVH (n = 187). Low-Fit/No LVH was used as the reference group for all survival analyses.

### 2.5 Statistical Analysis

Follow-up time is shown as mean (SD) and median years. Mortality rate was calculated as the ratio of events by the person-years of observation. Continuous variables are presented as mean (SD) values and categoric variables as relative frequencies (percentage). Baseline associations between categorical variables were tested using χ^2^ analysis.

One-way analysis of variance was used to evaluate mean differences of normally distributed variables across fitness/LVH categories. We tested the assumption of the equality of variances between groups by the Levene test and the assumption of normality with probability-probability plots. Post hoc procedures (Bonferroni) were performed for multiple comparisons.

Cox proportional hazard models were constructed to estimate hazard ratios (HRs) and 95% confidence intervals (CIs) for all-cause mortality across the fitness/LVH categories. The Low-Fit/No LVH category was used as the reference group. The analysis was adjusted for age, body mass index (BMI), hypertension, smoking and medications including angiotensin converting enzyme inhibitors (ACE-Is), angiotensin receptor blockers (ARBs), beta blockers (BBs), calcium channel blockers (CCBs), diuretics, oral hypoglycemic agents and statins.

The assumption of proportionality for all Cox proportional hazard analyses was graphically tested and fulfilled the criteria. All hypotheses were two-sided and *p < *0.05 was deemed statistically significant. All statistical analyses were performed using SPSS 19.0 software (SPSS Inc., Chicago, IL, USA).

## 3. Results

The flowchart of the population selection process is shown in Fig. 1. During the follow-up period of 24 years (median 8.9 years), there were 346 deaths, for an annual mortality rate of 4.3% in the entire cohort. The demographic characteristics and exercise data are presented in Table 1.

**Table 1.** **Demographic and clinical characteristics of type 2 diabetes subjects**.

	Entire cohort	Low Fit/No LVH	Low Fit/LVH	Fit/No LVH	Fit/LVH	*p* value
Participant’s n (%)	866	225 (26%)	236 (27%)	218 (25%)	187 (22%)	-
Age (years)	61.6 ± 9.9	65.2 ± 8.8	64.5 ± 8.5	57.6 ± 9.7	58.2 ± 10.0	<0.001
BMI (kg/m^2^)	30.1 ± 5.5	28.8 ± 5.0	31.4 ± 6.2	29.0 ± 4.6	31.4 ± 5.3	<0.001
Peak METs*	6.1 ± 1.8	4.7 ± 0.8	4.8 ± 0.7	7.8 ± 1.3	7.4 ± 1.2	<0.001
Resting heart rate (bpm)	74.4 ± 14.1	74.3 ± 14.0	75.2 ± 14.3	73.6 ± 14.0	74.3 ± 14.1	0.692
Resting systolic blood pressure (mmHg)	138.4 ± 22.1	140.3 ± 22.5	142.9 ± 23.2	132.7 ± 21.0	137.1 ± 20.1	<0.001
Resting diastolic blood pressure (mmHg)	79.5 ± 12.5	78.7 ± 12.5	79.8 ± 13.5	78.3 ± 11.9	81.6 ± 11.7	0.041
Cardiovascular disease (%)	56.6	62.2	62.3	45.0	56.1	0.13
Hypertension (%)	94.0	92.9	97.5	91.3	94.1	0.698
Dyslipidemia (%)	37.4	33.8	34.7	38.5	43.9	0.026
Smoking (%)	30.3	32.9	26.7	33.5	27.8	0.587
Aspirin (%)	8.0	6.2	6.8	9.2	10.2	0.091
ACE-I/ARBs (%)	32.7	29.3	26.7	39.9	35.8	0.02
Beta-blocker (%)	16.7	14.2	17.4	15.1	20.9	0.144
Calcium channel blocker (%)	20.4	18.2	18.2	18.3	28.3	0.021
Diuretics (%)	19.3	16.9	20.3	19.7	20.3	0.421
Statins (%)	11.8	8.4	8.5	17.0	13.9	0.010
Oral hypoglycemic agents (%)	29.6	28.0	30.9	26.6	33.2	0.474

*Continuous data are presented as mean ± SD, and categoric data as percentages. *p* values are for comparisons across groups. *1 MET = 3.5 mL O_2_/kg/min. BMI, body mass index; METs, metabolic equivalents; ARBs, angiotensin receptor blockers; ACE-I, angiotensin converting enzyme inhibitor.

At baseline, the mean age for the entire cohort was 61.6 ± 9.9 years. Approximately 94% of the study participants had hypertension. Fit individuals with or without LVH were younger compared to their Low-Fit counterparts. BMI levels were lower in those with No LVH, compared to individuals with LVH, regardless of fitness status.

Fit individuals were more likely to have dyslipidemia (*p* = 0.026) and to be treated with ACE-Is/ARB, and statins regardless of LVH status, whereas BB use was higher in individuals with LVH regardless of fitness status, but without statistical significance. Finally, Fit individuals with LVH were more likely to be treated with CCB (*p* = 0.021), compared to those in all other categories (Table 1). The use of aspirin was similar among all groups (Table 1). A minority of patients were treated with oral hypoglycemic agents (Table 1).

In a fully-adjusted model multivariate Cox proportional hazards analysis for the entire cohort revealed that age (HR: 1.032 (95% CI 1.019–1.045, *p* < 0.001)) and smoking (HR: 1.54 (95% CI 1.23–1.94, *p* < 0.001)) were strong predictors of mortality. CRF was inversely related to mortality risk. The adjusted mortality risk was 17% lower for every 1-MET increase in exercise capacity (HR: 0.83 (95% CI 0.78–0.91, *p* < 0.001)).

**Fig. 2.**
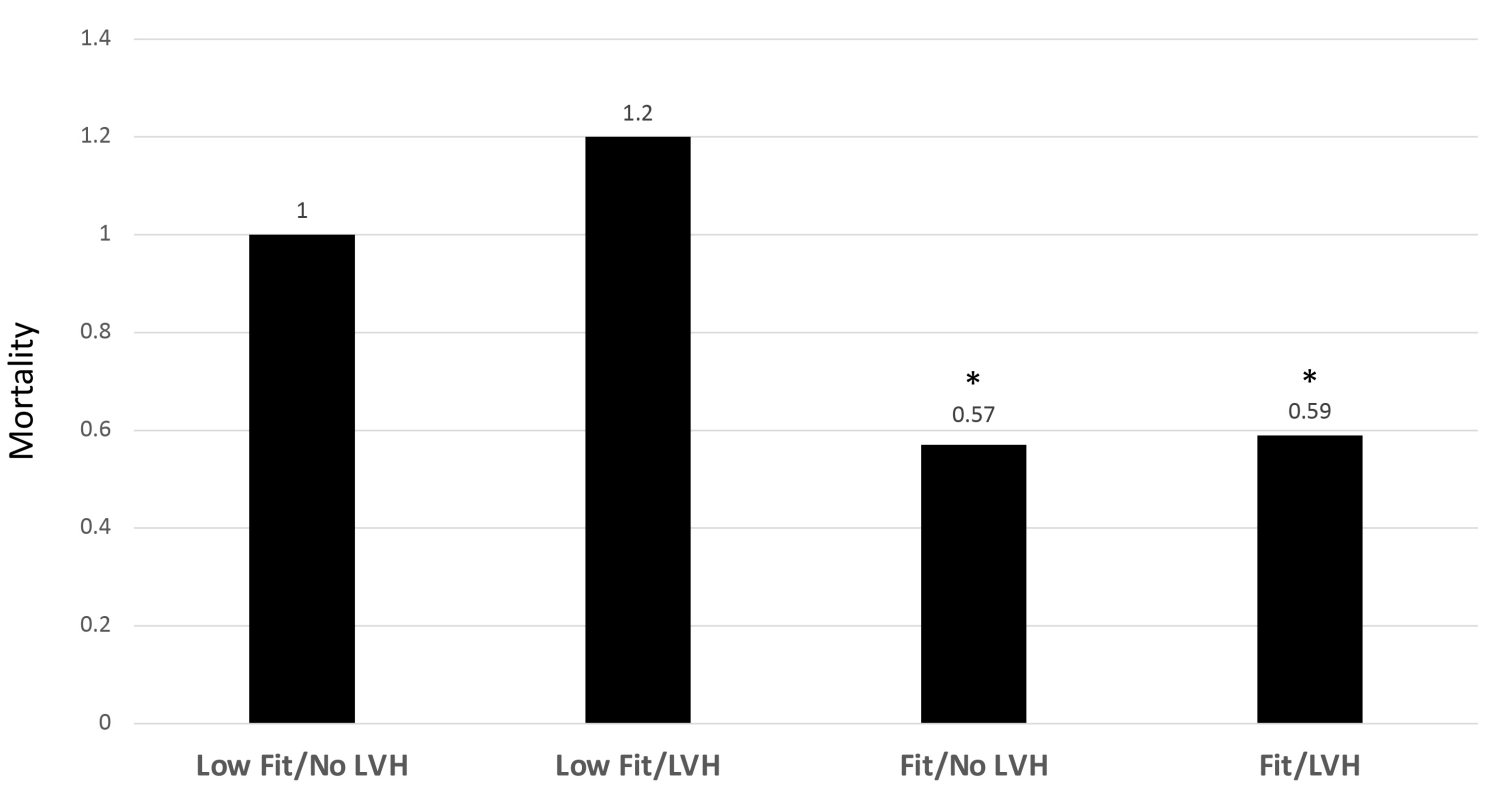
**Risk of mortality according to fitness and LVH categories in type 2 diabetes**. * *p* value = 0.002. LVH, left ventricular hypertrophy.

We also assessed mortality risk across the CRF/LVH categories with the Low-Fit/No LVH group as the referent. In a fully adjusted model mortality risk for Low-Fit individuals with LVH (Low-Fit/LVH group) was a 20% higher (HR: 1.20; 95% CI: 0.93–1.56, *p* = 0.15). For Fit individuals with and without LVH the mortality risk was 43% (HR: 0.57; 95% CI: 0.40–0.81, *p* = 0.002) and 41% (HR: 0.59; 95% CI: 0.42–0.82, *p* = 0.002) lower, respectively (Fig. 2).

## 4. Discussion

The findings of the current study are in accordance with previous reports that LVH increases the risk of mortality. In addition, we noted a strong, inverse and independent association between CRF and risk of all-cause mortality in men with T2DM with and without LVH. Although this association has been reported in both healthy and diseased populations [26,27,28,29,39,40], the unique aspect of the current study is that the increased mortality risk associated with LVH may be modulated by improved CRF. Moreover, the approximately 60% lower mortality risk in those with LVH was noted in those with CRF of 7.4 ± 1.2 METs. This CRF level is achievable by most middle-aged and older individuals by adhering to the well-accepted American College of Sports Medicine and American Diabetes Association physical activity guidelines of ≥150 minutes of moderate-intensity exercise per week [41,42].

Diabetic cardiomyopathy is a microvascular complication involving LVH in the absence of coronary artery disease and hypertension, and is an independent predictor of CVD [43,44,45]. In diabetes, LVH develops at an early stage, prior to the development of coronary artery disease or heart failure, due to abnormal myocardial energy metabolism associated with mitochondrial dysfunction—a process linked to insulin resistance and hyperinsulinemia [20,21,46,47,48,49,50]. Using robust measures of insulin sensitivity, adolescents with early onset T2DM have been shown to have reduced CRF and LVH which correlate with insulin resistance when compared to lean and obese controls [21]. Similarly, our cohort represented subjects with a recent onset of T2DM.

Although extensive discussion of the mechanisms involved in exercise-related favorable effects on cardiac structure and function is beyond the scope of this study [51], exercise appears to impact pathological cardiac hypertrophy through improvement in blood pressure and circulating factors such as aldosterone, angiotensin II, catecholamines, and natriuretic peptides [52]. Of note, LV mass decreased by 12% after 4 months of low-intensity exercise in subjects with severe hypertension [30]. Self-reported modest physical activity (>30 min twice per week) in patients with LVH in the LIFE study was associated with significant reductions of cardiovascular death [53]. Additional mechanistic links include inflammation and endothelial function: Inflammation (e.g., IL-6) has been independently linked to both diabetes and poor systolic function [54]. Moreover we propose that improved CRF, which is known to be associated with reduced inflammation, may be a mechanistic link. Another mechanistic connection regards endothelial dysfunction. Impaired endothelial function has been demonstrated in individuals with prediabetes and diabetes and endothelial dysfunction has adverse cardiovascular remodeling properties in diabetes [55]. Improved CRF via exercise improves endothelial aspects such as endothelial nitric oxide production [56].

These findings reinforce the public health benefits of CRF in individuals with T2DM and LVH and support the concept that it should be given as much attention by clinicians as other major risk factors. Healthcare professionals should discuss and promote this important finding with their diabetic patients who have LVH to initiate and maintain a physically active lifestyle.

## 5. Study Limitations

In addition to the inherent limitation of a retrospective study design, this study included only male veterans and the findings may not apply to women [57]. In addition, information regarding LVH and CRF status was available only at baseline, precluding the evaluation of changes in either factor during the follow-up period. Recent evidence, however, suggests that CRF change parallel mortality change and is therefore an independent mortality determinant [58]. Moreover, CRF is likely to decrease in more individuals than increase over time; In a large cohort of more than 93,000 we reported that CRF decreased in approximately 46% participants and increased in approximately 29% of the participants beyond the age-related decline, during a follow-up time of 5.8 ± 3.7 years [58]. This argues in favor of the need to increase our efforts in promoting physical activity for all ages. Finally, the sample size is relatively small, and more studies are needed to verify our findings.

## 6. Conclusions

Low-Fit individuals with LVH showed a trend toward higher mortality risk. This risk was significantly reduced in individuals with moderate fitness (i.e., ≥6 METs), regardless of LVH status. Given the health benefits associated with CRF, health care professionals should increase their efforts in promoting moderate-intensity physical activity for all patients [59].

## Data Availability

The datasets used and analyzed during the current study are available from the corresponding author on reasonable request.
